# A Network Model for the Correlation between Epistasis and Genomic Complexity

**DOI:** 10.1371/journal.pone.0002663

**Published:** 2008-07-16

**Authors:** Rafael Sanjuán, Miguel R. Nebot

**Affiliations:** 1 Institut Cavanilles de Biodiversitat i Biologia Evolutiva, Universitat de València, València, Spain; 2 Departament de Genètica, Universitat de València, València, Spain; 3 Instituto de Biología Molecular y Celular de Plantas (CSIC-UPV), València, Spain; 4 Centro de Física Teórica de Partículas (CFTP), Instituto Superior Técnico, Lisboa, Portugal; 5 Istituto Nazionale di Fisica Nucleare (INFN), Sezione di Roma Tre, & Dipartimento di Fisica “Edoardo Amaldi”, Università degli Studi Roma Tre, Roma, Italy; Ecole Normale Supérieure de Lyon, France

## Abstract

The study of genetic interactions (epistasis) is central to the understanding of genome organization and evolution. A general correlation between epistasis and genomic complexity has been recently shown, such that in simpler genomes epistasis is antagonistic on average (mutational effects tend to cancel each other out), whereas a transition towards synergistic epistasis occurs in more complex genomes (mutational effects strengthen each other). Here, we use a simple network model to identify basic features explaining this correlation. We show that, in small networks with multifunctional nodes, lack of redundancy, and absence of alternative pathways, epistasis is antagonistic on average. In contrast, lack of multi-functionality, high connectivity, and redundancy favor synergistic epistasis. Moreover, we confirm the previous finding that epistasis is a covariate of mutational robustness: in less robust networks it tends to be antagonistic whereas in more robust networks it tends to be synergistic. We argue that network features associated with antagonistic epistasis are typically found in simple genomes, such as those of viruses and bacteria, whereas the features associated with synergistic epistasis are more extensively exploited by higher eukaryotes.

## Introduction

A consequence of genetic interactions is that the combined effect of two or more mutations often deviates from what can be expected by looking at each individual mutation [1], [2]. This deviation, termed epistasis, can be antagonistic or synergistic depending on whether the combined mutational effect is respectively lower or higher than expected under no genetic interaction [2]. For mutations affecting fitness, the expected non-epistatic fitness of a genotype carrying several mutations is calculated by multiplying the fitnesses of the single mutants, whereas for other traits, the non-epistatic model is not necessarily multiplicative [2]. Although it is obvious that network parts interact and hence, that epistasis must be widespread, the causes for systematic deviations towards one kind of epistasis or another are still poorly understood. Yet, such deviations should play a key role in many evolutionary processes, including the evolution and maintenance of sexual reproduction [3], diploidy [4], dominance [5], speciation [6], or the genetic deterioration of small populations [7].

Until recently, it was common view that epistasis tended to be null on average, with some genes interacting synergistically, some antagonistically, and most in a non-epistatic fashion. Further, generalities about the average sign of epistasis were hampered by the apparently contradictory results obtained from different model organisms and by the variety of methodologies employed. However, a general correlation between epistasis and genomic complexity has been recently shown [8]. This correlation is such that in simple genomes as those of viruses and probably, some bacteria, epistasis tends to be antagonistic, whereas there is no apparent deviation from multiplicativity in unicellular eukaryotes and a transition towards synergistic epistasis occurs in higher eukaryotes. Recent advances in the characterization of molecular networks and in network theory provide new avenues for exploring the basis of epistasis and its relationship to complexity [9]–[11].

<1?tlsb=-.015w?>Evolutionary simulations [12], work with digital organisms [13], RNA folding studies [13], [14], data from mutagenized bacterial proteins [15], and quantitative trait loci analyses [16] have shown that epistasis correlates with mutational robustness. Therefore, we can hypothesize that the mechanisms responsible for robustness might also be relevant to epistasis. Several mechanisms of robustness have been identified. First, genetic redundancy can significantly reduce the impact of knock-out mutations, as shown in yeast [17], animals [18], [19], and plants [20]. Second, networks can compensate for failures by systemically adjusting the flow of matter to accommodate perturbations [21]. Finally, robustness can be achieved by embedding functions in autonomous protein complexes, transduction or transcriptional pathways, or differentiated cell types [22].

To assess whether the above mechanisms can also control the sign and intensity of epistasis, we simulated networks in which the number of nodes, functions and pathways, as well as the amount of multi-functionality, connectivity, and redundancy could be easily manipulated. Basic networks were configured as nodes connected by oriented edges, and pathways were defined as series of connected nodes. Mutations that knocked-out specific nodes were introduced and the ability of the network to accommodate these mutations was evaluated. We first present a simple model in which epistasis, calculated on a multiplicative basis, is null on average. We then add multifunctional nodes, extra edges, or redundant nodes to the model to test their effects on epistasis and robustness.

## Results

### Null model

**Figure 1:**
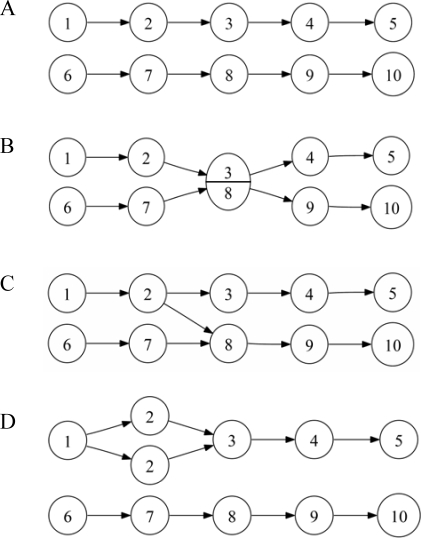
Graphical representation of two-pathway networks. Circles represent nodes, arrows their connections, and numbers the functions performed by the nodes. Nodes 1 and 6 are input nodes and nodes 5 and 10 are output nodes. A) The simplest case, or null model, constituted by two separate pathways of length five each. B) An example of multi-functionality: functions 3 and 8 are collapsed into a single node, which implies that a mutation at this node will inactivate both pathways. These two functions are physically linked but otherwise independent, meaning that the flow through the two pathways remains separated. C) Increased connectivity, through the addition of an extra edge. The extra edge between nodes 2 and 8 implies that the output produced by node 2 enters the lower pathway. Thus, mutations occurring at the lower pathway upstream of node 8 have no effect, provided the upper pathway is not mutated upstream of node 3. D) An example of redundancy: node 2 is duplicated, making single mutations at this node silent. A second mutation at node 2 has a 50% probability of being silent, depending on whether it hits the previously damaged copy or the intact one.

Similar to the “beanbag” model for genes [23], consider a “bag of functions”, where the term bag indicates the absence of epistasis. Each mutation knocks-out a function and the performance of the system declines proportionally to the number of functions available. Then, performance declines multiplicatively with the number of mutations. Specifically, being *n* the number of functions and *m* the number of mutations, the decline in performance, *W*, can be described by equation *W_m_* = (1−1/*n*)*^m^*. Deviations from this multiplicatively expected value would indicate the presence of epistasis. In terms of networks, the equivalent of the beanbag model is a system composed by non-overlapping pathways, each with the same number of linearly connected nodes (Fig. 1A). Mutations knock-out nodes and the performance of the network is proportional to the number of pathways that remain functional (i.e. able to produce an output). For simplicity, we thereafter focus on the case of two pathways, thought the conclusions would remain valid for higher numbers of pathways.

In the two-pathway case, single mutations invariantly inactivate one of the two pathways and hence, reduce performance to *W* = 0.5. For double mutants, the expected *W* is 0.5^2^ = 0.25, whereas the observed *W* can be 0 if the two mutations hit different pathways, or 0.5 if they hit the same pathway. Since these two outcomes are equiprobable, the average observed performance equals the multiplicatively expectation and, consequently, average epistasis is null. It can be proven that the same result holds for more than two mutations and more than two pathways of equal length (Text S1). Table 1 shows the analytically obtained epistasis values for several mutation numbers in the two-pathway case. The goal of our subsequent analyses is to assess how epistasis changes upon the addition of unequal pathway lengths, multi-functional nodes, extra-edges, or redundant nodes to this null model.

**Table 1:** Analytically obtained epistasis values for various mutation numbers in the simple, ten-node two-pathway model (Figure 1A) for equal (5+5) and unequal (8+2) path lengths.

		Equal path lengths (5+5)			Unequal path lengths (8+2)		
Mutation number	Possible epistasis values	Frequency	Mean	Variance	Frequency	Mean	Variance
2	−0.250	50.0%	0	0.250	32.0%	0.090	0.234
	0.250	50.0%			68.0%		
4	−0.063	87.5%	0	0.165	58.9%	0.143	0.246
	0.438	12.5%			41.1%		
7	−0.008	98.4%	0	0.062	79.0%	0.097	0.204
	0.492	1.6%			21.0%		
10	−0.001	99.8%	0	0.022	89.3%	0.053	0.155
	0.499	0.2%			10.7%		

The case of equal path lengths corresponds to the null model (see text). For each mutation number, the two possible epistasis values, the frequency of each, mean epistasis and its variance are shown. Analytical results were cross-checked against simulations to confirm the consistency of the two approaches (not shown).

### Unequal pathway lengths

When one of the two pathways is extended at the expense of the other, the first mutation still reduces the fraction of active outputs to 0.5, but after a second mutation, the outcomes *W* = 0 and *W* = 0.5 are not equally likely anymore. The chances that both mutations hit the same pathway are higher and therefore, average epistasis becomes antagonistic. This result can also be proven for two or more mutations and two or more pathways (Text S1).

Since unequal rather than equal path lengths constitute the general case, simple, unreticulated, networks should exhibit a tendency towards antagonistic epistasis, as also suggested by other network models [24]. Deviation from multiplicativity occurs because mutations often hit essential parts of the same pathway. This multiple-hit effect is important for networks with a small number of pathways, but vanishes as the number of pathways increases. For instance, for two pathways of lengths two and eight, two mutations have a 68% chance of hitting the same pathway, whereas for 20 pathways, 10 of length two and 10 of length eight, this probability is only 6.8%. Multiple-hitting can thus potentially explain antagonistic epistasis in small genomes. Work with simple digital organisms confirms this prediction [25].

### Multifunctional nodes

**Figure 2:**
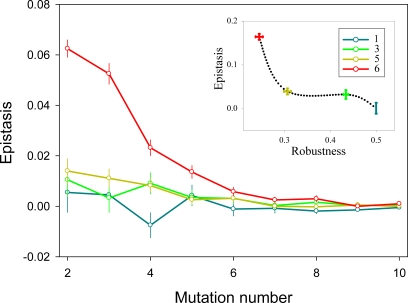
Effect of multi-functionality on epistasis in two-pathway networks. The 10 functions of the network were assigned to 10, 7, 5, or 4 nodes. Each node performed thus 1, up to 3, up to 5, or up to 6 functions, respectively, as shown in the color legend. For each mulfi-functionality value, 1000 networks were created, choosing multifunctional nodes at random. For each network, we introduced from 1 to 10 mutations. The main graph reports average epistasis values±SEM. The inset shows the correlation between robustness and total epistasis. Total epistasis was computed by summing all epistasis coefficients for mutation numbers larger than 1. Robustness was calculated as detailed in the text.

To construct integrated networks, we first allowed pathways to share nodes, such that some nodes became multifunctional. To do so, the number of nodes in the two-pathway network was reduced without changing the total number of functions (Fig. 1B). Mutational analysis of these networks showed that, as multi-functionality increased, there was a shift from multiplicative effects towards antagonistic epistasis (Fig. 2). This is in agreement with previous work with digital organisms, showing that a shift from antagonistic epistasis towards multiplicativity can be obtained when some tasks are removed from genomes without varying genome length [26]. Notice that our definition of multi-functionality is related to the more general notion of modularity.

Finally, we observed that robustness, calculated as the fraction of pathways that remains active upon the introduction of single mutations, dropped as epistasis became more antagonistic. This loss of robustness occurs because, as the number of multifunctional nodes increases, there is a higher chance that single mutations simultaneously damage several pathways (pleiotropy).

### Connectivity

**Figure 3:**
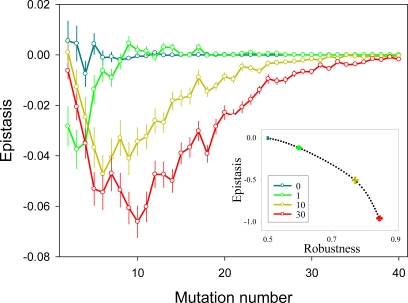
Effect of connectivity on epistasis in two-pathway networks. Increasing numbers of extra edges (0, 1, 10, 30) were allowed (color legend), which consequently increases connectivity (*c* = 0.8, 0.9, 1.8, and 3.8, respectively). For each connectivity value, 1000 networks were created, choosing extra edges at random. For each network, 1 to 40 mutations were introduced. The main graph reports average epistasis values±SEM. The inset graph shows the correlation between robustness and total epistasis. Total epistasis and robustness were computed as detailed in Fig. 2 legend.

Connectivity in metabolic networks can arise from the use of universal metabolites (e.g. ATP, or acetyl-coenzyme A), or from the existence of interchangeable regulatory elements which participate in different pathways. Similar to what usually happens *in silico* [27], [28], connectivity in biological networks allows the flow of matter to be shuttled throughout the system, making it more stable to perturbations [21], [29]. We introduced extra edges between pairs of nodes to increase the connectivity (*c*) of the network (Fig. 1C). These extra edges make it possible that the flow throughout the mutated pathways is restored from another pathway and thus, that some mutations are silent. As expected, we observed that the effect of single mutations decreased with increasing connectivity. Moreover, this trend was accompanied by a shift from multiplicativity to synergistic epistasis (Fig. 3). Interestingly, for larger connectivity values, synergistic epistasis peaked at larger mutation numbers. For example, for *c* = 0.9 synergism was statistically not significant anymore beyond 8 mutations, whereas for *c* = 2.8 it remained so for up to 37 mutations. The reason for this difference is that as connectivity increases, the network can buffer more mutations and, as long as mutations are silent, no epistasis is produced. However, as the number of mutations increases, the buffering ability of the network is exhausted and synergistic epistasis is released.

### Redundancy

**Figure 4:**
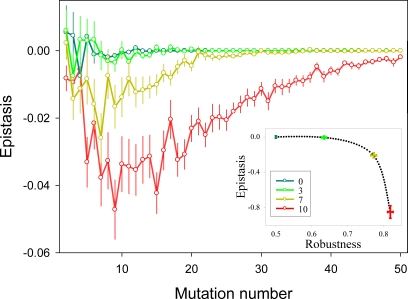
Effect of redundancy on epistasis in two-pathway networks. Increasing numbers of duplicates (0, 3, 7, 10) were introduced (color legend). For each redundancy level, 1000 networks were created, choosing duplicates at random (nodes could be duplicated more than once). For each network, 1 to 50 mutations were introduced. The main graph reports average epistasis values±SEM. The inset graph shows the correlation between robustness and total epistasis. Total epistasis and robustness were calculated as detailed in Fig. 2 legend.

In biological systems, redundancy arises from gene duplications or polyploidizations. Large-scale analyses have shown that genetic redundancy plays a role in buffering mutational damage in yeast [17], animals [18], [19], and plants [20]. Redundancy was incorporated into our network model by introducing identical copies of the existing nodes, such that the duplicates conserved their position in the network and all the connections with other nodes (Fig. 1D). Mutations at redundant nodes were silent if at least one of the copies of the node remained non-mutated. The robustness of the two-pathway network obviously increased as redundancy increased. This trend was, again, accompanied by a shift from multiplicativity to synergistic epistasis (Fig. 4). However, low levels of redundancy were not enough to produce significant amounts of synergism, in contrast to the results obtained in the above analysis of connectivity. For levels up to 50%, redundancy had a much weaker effect on epistasis than on robustness. For higher levels of redundancy, in contrast, strong synergistic epistasis was generated.

Whereas redundancy decreases the probability that mutational effects are expressed, such buffering becomes necessarily less efficient as mutations accumulate. This is why redundancy is not only a source of robustness, but also a source of synergistic epistasis. A clear-cut example is that of synthetic lethality, whereby inactivation of each of two duplicate genes produces little or no visible effect whereas the double mutant is non-viable [30]. According to our results, though, high levels of redundancy are needed to produce significant amounts of synergism.

## Discussion

According to theory, selection should favour the evolution of developmental and somatic processes that increase genetic robustness in small populations with long generation times, whereas in large, rapidly replicating populations, lack of robustness should be selected [31]. These considerations lead us to expect that higher eukaryotes should to be more robust to mutation than viruses or bacteria and, as long as the correlation between robustness and epistasis holds, these differences would translate into differences in epistasis. Also, from a more molecular perspective, divergent paralogs, genome-scale duplications, polyploidizations, dominance, alternative pathways in metabolic networks, or multiple regulatory elements per gene are forms of complexity which have been shown to, or can be assumed to confer robustness [17]–[22].

These variegate molecular mechanisms can be assigned to more general features, as redundancy, multi-functionality, and connectivity. As a sum up of our results, we have shown that simple, unreticulated, networks with few pathways tend to display antagonistic epistasis due the so-called multiple-hit effect, a tendency that becomes more marked when multifunctional nodes are frequent. In contrast, increased connectivity and redundancy produce synergistic epistasis. Our model captures very basic features of networks and thus, these conclusions might be valid for many kinds of networks. However, it still needs to be elucidated whether these general features are differentially found in genomes of increasing complexity. A few inklings are discussed below.

Several RNA genomes have been shown to display antagonistic epistasis, including those of viroids [14], bacteriophage [32], negative-stranded mammalian viruses [33], and retroviruses [34]. Some data suggest that epistasis might also tend to be antagonistic in bacteria [35]. RNA genomes encode few genes, are highly compact and show a high degree of multi-functionality, which results in marked fitness tradeoffs [36], [37]. In the light of our results, these features would also explain their tendency to exhibit antagonistic epistasis. This is also a likely scenario for ancestral metabolic networks, probably constituted by few enzymes with broad specificities [38]. In the case of bacteria, an *in silico* study of the global transcriptional regulatory network of *Escherichia coli* indicated that genes can be grouped in few independent functional groups [39], though it remains to be clarified whether this number is low enough for multiple-hit to generate a significant amount of antagonistic epistasis.

A recent extensive analysis of the enzymatic complement in different genomes indicates that the ratio of the number of enzymatic functions performed by the organism to the number of genes encoding these functions is higher in prokaryotes than in eukaryotes [40]. These differences can be explained either in terms of redundancy or multi-functionality. However, interestingly, the ratio was higher than one in some bacteria, which would unambiguously indicate multi-functionality. Finally, it seems reasonable that, as a general rule, large genomes as those of higher eukaryotes should have more room for subdividing genetic information into partially independent subsets, a subdivision that would be further facilitated by subcellular compartmentalization and cellular differentiation [41].

Functional connectivity in biological networks allows the flow of matter to be shuttled throughout the system, making it more stable in the face of perturbations [21], [29]. This ability, termed distributed robustness, has been described in processes as diverse as the chemostatic responses of bacteria or the self-regulatory behaviour of cortical neurons [28]. Distributed robustness seems pervasive in *Caenorhabditis elegans*, as shown by the fact that 90% of its single-copy genes can be silenced without producing any detectable phenotypic effect [18]. It also seems to be important for mammals, as suggested by phylogenetic analyses comparing human and rodent genomes [42]. Although it is at present difficult to correlate organismal complexity with network connectivity, this correlation is suggested by the observation that, as the number of genes in the genome increases, there is a disproportionate increase in the number of transcription factors [43], [44].

Finally, gene duplication and the evolving subtle differences between paralogs are thought to be a major source of biological complexity [45], [46]. Despite large gene families exist in bacteria, the number of duplicates and the size of gene families increase in eukaryotes [47]. Further, the amount of paralogs is not evenly distributed among the latter. In yeast, 13% of the genome is thought to be a relic from an ancestral whole genome duplication event [48], whereas in higher eukaryotes, the number of duplicates increases. Gene duplication primarily produces redundancy, but it can also reduce multi-functionality if the duplicates diverge and subfunctionalize. In the first case, gene duplications would tend to produce synergistic epistasis, whereas in the second, they would simply relax antagonistic epistasis. We have shown, though, that high levels of redundancy are required to produce significant synergistic epistasis. We can thus speculate that, whereas in prokaryotes and lower eukaryotes the number of extant duplicated genes might not be high enough to generate observable levels of synergistic epistasis, it could be so in higher eukaryotes.

## Methods

### Model

#### Construction of the network

The pathways of the network were encoded by a vector *v_0_* with as many entries as there are nodes, in which we assigned entries *n_i_* = 1 to the input nodes and *n_i_* = 0 to the rest of them. The position of input nodes within this vector was random, such that the resulting spectrum of pathway lengths followed a uniform distribution of integers within a user-defined range [ℓ_min_; ℓ_max_]. Whenever the sum of pathway lengths was not equal to the overall length *n*, some small reshuffling of lengths was applied to do so. The resulting average pathway length is 2*n*/(ℓ_min_+ℓ_max_).

The edge-connecting structure of the network was represented by a square matrix *M* encoding the existing connections among the *n* nodes. For each pair of connected nodes *n_i_*→*n_j_*, *M*(*n_i_*,*n_j_*) = 1, otherwise *M*(*n_i_*,*n_j_*) = 0. Upon calculation of *v_1_* = *Mv_0_*, non-zero entries in *v_1_* indicate the transmission of the input throughout the network. Pathway ends were represented by diagonal matrix entries *M*(*n_k_*,*n_k_*) = 1. This stopped the propagation of node activation and retained the information of an active pathway output. The iterative application of *M* becomes redundant as soon as a stable pattern of zero/non-zero entries is reached.

For simplicity, data shown correspond to networks encoding 10 functions divided into two pathways. Similar results were obtained with larger networks (50 and 100 functions) as well as with larger numbers of pathways.

#### Functions, edges, and nodes

Formally, each function is a column or row in the square matrix *M*. Each non-zero element in *M* defines an edge. Finally, if there is no multi-functionality, each node is just the equivalent of one function, whereas if there is multi-functionality, a number of functions are assigned to each node. This latter information is external to the matrix *M* and therefore, nodes do not affect its algebra. In biological terms, a node would be a gene in a genetic network or an enzyme in a metabolic network. Nodes are the target of mutations (all functions in the node are simultaneously mutated). Graphically, each number in Fig. 1 is a function, each arrow is an edge, and each circle is a node. For instance, two redundant nodes have the same number because they have the same function, and multi-functional nodes have several numbers because they perform several functions.

#### Multi-functionality, connectivity, and redundancy

To model node multi-functionality (i.e. the case in which there are more functions than nodes), the dimensions of both *M* and *v_0_* were increased through the insertion, where appropriate, of new columns and rows in the case of *M*, and just rows in the case of *v_0_*. Those new *n′* elements were randomly assigned to the basic *n* nodes. Each node could be linked to more than one new element. Given this configuration, *n* is the number of nodes and *f* = *n*+*n′* the number of functions performed by these nodes. Multi-functionality (*m*) can be measured as the probability that a node performs more than one function, *m* = 1−(1−1/*n*)*^f^* ^−*n*^.

Extra edges (also oriented) are straightforwardly incorporated into the structure matrix *M*: for each connected pair *n_i_*→*n_j_*, one sets *M*(*n_i_*,*n_j_*) = 1. Since *M* is a representation of functions, edges linked functions instead of nodes. In other words, for multifunctional nodes, edges were wired independently for each function. This allowed us to separate the effects of connectivity from those of multi-functionality. Extra edges between nodes of the same pathway were allowed but, given any edge *n_i_*→*n_j_*, the condition *i*<*j* was imposed to avoid loops. The number of extra edges per node was drawn at random, producing roughly a Poisson distribution for the number of edges departing from a node. We also explored the case of a power law distribution, which is probably a more natural one [49] and the results were qualitatively similar (not shown). Connectivity (*c*) was calculated as the average number of edges departing from a node.

Finally, in terms of modelling, redundancy is dealt together with mutations and thus no per se modification of the up-to-now explained framework is required.

#### Mutational analysis of the network

Mutations occurred randomly and with replacement, that is, all nodes had the same chance of being mutated and each node could be mutated more than once. All mutated nodes were knocked-out, with no intermediate mutational effects. We expect that assigning intermediate effects to mutations would only add noise to the results. From the list of candidate mutations, we determined the effective ones. First, if redundancy was present, mutations acting on redundant nodes were randomly distributed among the different copies. Mutations hitting redundant nodes were silent provided at least one of the copies remained mutation-free. Second, as mentioned above, a mutation hitting a multifunctional node affected all functions associated to the node. Once the list of effective mutations was obtained, the columns of *M* corresponding to these positions were set to zero. One can automatically set the *i* ^th^ column of *M* to zero by right multiplication with an identity matrix in which the *i* ^th^ diagonal is replaced by 0; the corresponding matrix encoding all the effective mutations is denoted M*. The last step is the calculation of active pathways. Once the stable state *v_final_* = (MM*)^k^ *v_0_* is reached, this fraction is easily read from the number of non-zero values in the entries corresponding to the output nodes of the different pathways.

Robustness was defined as the average fraction of pathways that remained active upon the introduction of single mutations. For epistasis (*ε*), we followed the standard mathematical definition 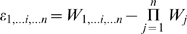 with *n*≥2 and *W* the fraction of successfully produced outputs [2].

Analytical results shown in Table 1 are derived in the supplementary Text S1.

All network models were generated with Mathematica (Wolfram Research).

## Supporting Information

Text S1 (0.08 MB PDF)
