# Supplementary material for: A Network Model for the Correlation between Epistasis and Genomic Complexity
Source: PLoS One. 2008 Jul 16;3(7):e2663. doi: 10.1371/journal.pone.0002663 (PMC2481279; doi:10.1371/journal.pone.0002663)
Supplement: Text S1 — (0.08 MB PDF) [file pone.0002663.s001.pdf]

ANALYTICAL CALCULATIONS

Analytical calculation of expected epistasis values provides an important cross check of the correctness of the numerical procedure. In spite of just involving combinatorics, calculations in full fledged situations – i.e. including multifunctional nodes, connectivity and redundancy – are beyond our present aims and thus we concentrate on the simpler scenario in which they are absent. Within that assumption this appendix presents an organised and systematic approach to such analytical calculations; beside the general scheme, several exhaustively detailed examples are worked out for clearness' sake.

The general recipe

I. Each class of equivalent mutational situation is represented univocally by a pattern of *boxes* determined according to the following rules.

1. Overall, there are as many boxes as mutations.
2. The complete pattern may contain several subpatterns. There can be as many of them as different lengths of pathways are considered. Each subpattern corresponds to a different pathway length.
3. Each subpattern is an arrangement of boxes in rows and columns such as the number of boxes in a row (or column) cannot increase when we move down (or right) within it.

   Example: those are allowed subpatterns 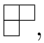, 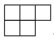, 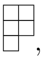, 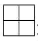; those are forbidden subpatterns 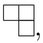, 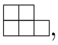, 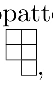, 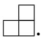.
4. The number of columns in a given subpattern is limited to the number of different pathways in the considered network having the length associated to this subpattern.

II. The number of different cases in a given class of mutations is derived from the corresponding pattern according to the following rules.

1. The overall number associated to a pattern is obtained as the product of the numbers corresponding to the subpatterns it is made of.
2. The number corresponding to a subpattern associated to paths of length n when k pathways of this length are present is calculated as follows:
   (a) To each box in the first (top) row a factor $n(k+1-c)$ is associated, where c is the column on top of which the box is found (i.e. a factor nk for the top left box, $n(k-1)$ for the box next to it on the right and so on).
   (b) To each box not in the first row is associated a factor of n.
   (c) A factor $m!$ where m is the number of boxes in the subpattern.
   (d) The product of the factors a), b) and c) is then divided by the symmetry factor of the subpattern, which is obtained as the product of the following factors

- $\prod_c (p_c!)$, where p_c is the number of boxes in column c
- $\prod_c (q_c!)^{1/q_c}$, where q_c is the number of columns with the same number of boxes as column c (including column c). The power $1/q_c$ appears to cancel the overcounting coming from the product being extended to all columns, for example, for three identical columns one would get $(3!)^{1/3}(3!)^{1/3}(3!)^{1/3} = 3!$.

In plain words, the *symmetry* factor has a piece coming from the permutation symmetry of boxes in each column and a piece coming from the permutation symmetry of identical columns.

III. The complete analysis for a given number of mutations is obtained by considering the contribution of *all the possible allowed patterns.*

1. The number of cases in each class is computed as in II.
2. The total number of possible cases with g genes and m mutations is just g^m.
3. The epistasis value associated to each pattern is straightforward to compute as the number of columns directly gives the number of disabled paths. The contribution to the final epistasis value is then weighted by the number of cases.
4. Knowing the expected epistasis value from the previous item the expected variance is also straightforward to compute.

Let us illustrate the procedure with some examples.

The examples

Example 1
100 nodes, 10 paths of (equal) length 10, 5 mutations.
Total number of cases: $100^5 = 10^{10}$.
Patterns:

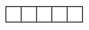

$$\mapsto 100 \times 90 \times 80 \times 70 \times 60 \times \frac{5!}{5!} = 3024 \times 10^6$$

$$W = 5/10; \qquad w = 9/10; \qquad \epsilon = \frac{5}{10} - \left(\frac{9}{10}\right)^5 = -\frac{9049}{10^5}$$

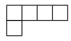

$$\mapsto 100 \times 90 \times 80 \times 70 \times 10 \times \frac{5!}{2!\ 3!} = 5040 \times 10^6$$

$$W = 6/10; \qquad w = 9/10; \qquad \epsilon = \frac{6}{10} - \left(\frac{9}{10}\right)^5 = \frac{951}{10^5}$$

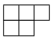

$$\mapsto 100 \times 90 \times 80 \times 10^2 \times \frac{5!}{2!\ 2!\ 2!} = 1080 \times 10^6$$

$$W = 7/10; \qquad w = 9/10; \qquad \epsilon = \frac{7}{10} - \left(\frac{9}{10}\right)^5 = \frac{10951}{10^5}$$

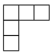

$$\mapsto 100 \times 90 \times 80 \times 10^2 \times \frac{5!}{3!\ 2!} = 720 \times 10^6$$

$$W = 7/10; \qquad w = 9/10; \qquad \epsilon = \frac{7}{10} - \left(\frac{9}{10}\right)^5 = \frac{10951}{10^5}$$

$$\ydiagram{2,2,1} \mapsto 100 \times 90 \times 10^3 \times \frac{5!}{3!\ 2!} = 90 \times 10^6$$

$$W = 8/10; \qquad w = 9/10; \qquad \epsilon = \frac{8}{10} - \left(\frac{9}{10}\right)^5 = \frac{20951}{10^5}$$

$$\ydiagram{2,1,1,1} \mapsto 100 \times 90 \times 10^3 \times \frac{5!}{4!} = 45 \times 10^6$$

$$W = 8/10; \qquad w = 9/10; \qquad \epsilon = \frac{8}{10} - \left(\frac{9}{10}\right)^5 = \frac{20951}{10^5}$$

$$\ydiagram{1,1,1,1,1} \mapsto 100 \times 10^4 \times \frac{5!}{5!} = 1 \times 10^6$$

$$W = 9/10; \qquad w = 9/10; \qquad \epsilon = \frac{9}{10} - \left(\frac{9}{10}\right)^5 = \frac{30951}{10^5}$$

Number of cases (check)

$$(3024 + 5040 + 1080 + 720 + 90 + 45 + 1) \times 10^6 = 10^{10}$$

Mean epistasis

$$\bar{\epsilon} = 10^{-10}(-9049 \times 30240 + 951 \times 50400 + 10951 \times (10800 + 7200) + \\ 20951 \times (900 + 450) + 30951 \times 10) = 0$$

Standard deviation

$$\sigma(\bar{\epsilon}) = \frac{10^{-2}}{\sqrt{10^{10} - 1}}(9049^2 \times 3024 + 951^2 \times 5040 + 10951^2 \times (1080 + 720) + \\ 20951^2 \times (90 + 45) + 30951^2)^{1/2} \simeq 0.0727$$

Example 2
100 nodes, 5 paths of length 12 and 5 paths of length 8, 3 mutations.
Total number of cases $100^3 = 10^6$.
Patterns (the superscript in each subpattern designates the length of the paths):

$$\ydiagram{3}^{12} \mapsto 5 \times 4 \times 3 \times 12^3 \times \frac{3!}{3!} = 103680$$

$$W = 7/10; \qquad w = 9/10; \qquad \epsilon = \frac{7}{10} - \left(\frac{9}{10}\right)^5 = -\frac{29}{10^3}$$

$$\ydiagram{3}^{8} \mapsto 5 \times 4 \times 3 \times 8^3 \times \frac{3!}{3!} = 30720$$

$$W = 7/10; \qquad w = 9/10; \qquad \epsilon = \frac{7}{10} - \left(\frac{9}{10}\right)^5 = -\frac{29}{10^3}$$

$$\square\square^{12}\ \square^{8} \mapsto 5 \times 4 \times 12^2 \times 5 \times 8 \times \frac{3!}{2!} = 345600$$

$$W = 7/10; \qquad w = 9/10; \qquad \epsilon = \frac{7}{10} - \left(\frac{9}{10}\right)^5 = -\frac{29}{10^3}$$

$$\square^{12}\ \square\square^{8} \mapsto 5 \times 12 \times 5 \times 4 \times 8^2 \times \frac{3!}{2!} = 230400$$

$$W = 7/10; \qquad w = 9/10; \qquad \epsilon = \frac{7}{10} - \left(\frac{9}{10}\right)^5 = -\frac{29}{10^3}$$

$$\begin{smallmatrix}\square\square\\ \square\end{smallmatrix}^{12} \mapsto 5 \times 4 \times 12^3 \times \frac{3!}{2!} = 103680$$

$$W = 8/10; \qquad w = 9/10; \qquad \epsilon = \frac{8}{10} - \left(\frac{9}{10}\right)^5 = \frac{71}{10^3}$$

$$\begin{smallmatrix}\square\square\\ \square\end{smallmatrix}^{8} \mapsto 5 \times 4 \times 8^3 \times \frac{3!}{2!} = 30720$$

$$W = 8/10; \qquad w = 9/10; \qquad \epsilon = \frac{8}{10} - \left(\frac{9}{10}\right)^5 = \frac{71}{10^3}$$

$$\begin{smallmatrix}\square\\ \square\end{smallmatrix}^{12}\ \square^{8} \mapsto 5 \times 12^2 \times 5 \times 8 \times \frac{3!}{2!} = 86400$$

$$W = 8/10; \qquad w = 9/10; \qquad \epsilon = \frac{8}{10} - \left(\frac{9}{10}\right)^5 = \frac{71}{10^3}$$

$$\square^{12}\ \begin{smallmatrix}\square\\ \square\end{smallmatrix}^{8} \mapsto 5 \times 12 \times 5 \times 8^2 \times \frac{3!}{2!} = 57600$$

$$W = 8/10; \qquad w = 9/10; \qquad \epsilon = \frac{8}{10} - \left(\frac{9}{10}\right)^5 = \frac{71}{10^3}$$

$$\begin{smallmatrix}\square\\ \square\\ \square\end{smallmatrix}^{12} \mapsto 5 \times 12^3 \times \frac{3!}{3!} = 8640$$

$$W = 9/10; \qquad w = 9/10; \qquad \epsilon = \frac{9}{10} - \left(\frac{9}{10}\right)^5 = \frac{171}{10^3}$$

$$\begin{smallmatrix}\square\\ \square\\ \square\end{smallmatrix}^{8} \mapsto 5 \times 8^3 \times \frac{3!}{3!} = 2560$$

$$W = 9/10; \qquad w = 9/10; \qquad \epsilon = \frac{9}{10} - \left(\frac{9}{10}\right)^5 = \frac{171}{10^3}$$

Number of cases (check)

$$103680+30720+345600+230400+103680+30720+86400+57600+8640+2560 = 10^6$$

Mean epistasis

$$\bar{\epsilon} = 10^{-6}(-\frac{29}{10^3}(103680 + 30720 + 345600 + 230400)$$
$$+ \frac{71}{10^3}(103680 + 30720 + 86400 + 57600)$$
$$+ \frac{171}{10^3}(8640 + 2560)) = \frac{27}{25000} = 0.00108$$

Standard deviation

$$\sigma(\bar{\epsilon}) = \frac{1}{\sqrt{10^6 - 1}}((-29/10^3 - \bar{\epsilon})^2(103680 + 30720 + 345600 + 230400)+$$
$$(71/10^3 - \bar{\epsilon})^2(103680+30720+86400+57600) + (171/10^3 - \bar{\epsilon})^2(8640+2560))^{1/2}$$
$$\simeq 0.0482$$

Example 3
10 nodes, 1 path of length 8 and 1 path of length 2, 7 mutations (see Table 2). Total number of cases 10^7.
Patterns (the superscript in each subpattern designates the length of the paths):

$$\begin{array}{c}\square\\\square\\\square\\\square\\\square\\\square\\\square\end{array}^{8} \mapsto 8 \times 8^6 \times \frac{7!}{7!} = 2097152$$

$$W = 1/2; \qquad w = 1/2; \qquad \epsilon = \frac{1}{2} - \left(\frac{1}{2}\right)^7 = \frac{63}{128}$$

$$\begin{array}{c}\square\\\square\\\square\\\square\\\square\\\square\\\square\end{array}^{2} \mapsto 2 \times 2^6 \times \frac{7!}{7!} = 128$$

$$W = 1/2; \qquad w = 1/2; \qquad \epsilon = \frac{1}{2} - \left(\frac{1}{2}\right)^7 = \frac{63}{128}$$

$$\begin{array}{c}\square\\\square\\\square\\\square\\\square\\\square\end{array}^{8} \square^2 \mapsto 8 \times 8^5 \times 2 \times \frac{7!}{6!} = 3670016$$

$$W = 0; \qquad w = 1/2; \qquad \epsilon = 0 - \left(\frac{1}{2}\right)^7 = -\frac{1}{128}$$

$$\square^8 \begin{array}{c}\square\\\square\\\square\\\square\\\square\\\square\end{array}^{2} \mapsto 8 \times 2 \times 2^5 \times \frac{7!}{6!} = 3584$$

$$W = 0; \qquad w = 1/2; \qquad \epsilon = 0 - \left(\frac{1}{2}\right)^7 = -\frac{1}{128}$$

$$\begin{array}{c}\square\\\square\\\square\\\square\\\square\end{array}^{8} \begin{array}{c}\square\\\square\end{array}^{2} \mapsto 8 \times 8^4 \times 2 \times 2 \times \frac{7!}{5!\ 2!} = 2752512$$

$$W = 0; \qquad w = 1/2; \qquad \epsilon = 0 - \left(\frac{1}{2}\right)^7 = -\frac{1}{128}$$

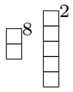 $$\mapsto 8 \times 8 \times 2 \times 2^4 \times \frac{7!}{2!\ 5!} = 43008$$

$$W = 0; \qquad w = 1/2; \qquad \epsilon = 0 - \left(\frac{1}{2}\right)^7 = -\frac{1}{128}$$

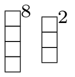 $$\mapsto 8 \times 8^3 \times 2 \times 2^2 \times \frac{7!}{4!\ 3!} = 1146880$$

$$W = 0; \qquad w = 1/2; \qquad \epsilon = 0 - \left(\frac{1}{2}\right)^7 = -\frac{1}{128}$$

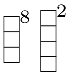 $$\mapsto 8 \times 8^2 \times 2 \times 2^3 \times \frac{7!}{3!\ 4!} = 286720$$

$$W = 0; \qquad w = 1/2; \qquad \epsilon = 0 - \left(\frac{1}{2}\right)^7 = -\frac{1}{128}$$

Number of cases (check)

$$2097152 + 128 + 3670016 + 3584 + 2752512 + 43008 + 1146880 + 286720 = 10^7$$

Mean epistasis

$$\bar{\epsilon} = 10^{-7}\left(\frac{63}{128}(2097152 + 128)\right.$$
$$\left. - \frac{1}{128}(3670016 + 3584 + 2752512 + 43008 + 1146880 + 286720)\right) = 0.09705$$

Standard deviation

$$\sigma(\bar{\epsilon}) = \frac{1}{\sqrt{10^7 - 1}}((63/128 - \bar{\epsilon})^2(2097152 + 128) +$$
$$(-1/128 - \bar{\epsilon})^2(3670016 + 3584 + 2752512 + 43008 + 1146880 + 286720))^{1/2}$$
$$\simeq 0.20356$$

N.B. Readers having some familiarity with number theory or the symmetric group S_n will recognise those box subpatterns as *Ferrers graphs* or *Young diagrams*; they are directly useful here because they represent partitions of integer numbers (in this case, partitions of the number of mutations), just what is required to have an organised and exhaustive procedure for our calculations.

SUPPLEMENTARY REFERENCES

1. A. L. Barabasi and R. Albert, *Science.* 286, 509-512 (1999).
2. J. B. Wolf, E. D. Brodie III, M. J. Wade, Epistasis and the evolutionary process (*Oxford University Press*, Oxford, 2000).
